# Sex-heterogeneous SNPs disproportionately influence gene expression and health

**DOI:** 10.1371/journal.pgen.1010147

**Published:** 2022-05-05

**Authors:** Michela Traglia, Margaux Bout, Lauren A. Weiss

**Affiliations:** Institute for Human Genetics, Department of Psychiatry and Weill Institute for Neurosciences, University of California, San Francisco, California, United States of America; University of Miami, Miller School of Medicine, UNITED STATES

## Abstract

Phenotypic differences across sexes are pervasive, but the genetic architecture of sex differences within and across phenotypes is mostly unknown. In this study, we aimed to improve detection power for sex-differentially contributing SNPs previously demonstrated to be enriched in disease association, and we investigate their functions in health, pathophysiology, and genetic function. We leveraged GIANT and UK Biobank summary statistics and defined a set of 2,320 independent SNPs having sexually dimorphic effects within and across biometric traits (MAF > 0.001, *P* < 5x10^-8^). Biometric trait *sex-heterogeneous SNPs (sex-het SNPs)* showed enrichment in association signals for 20 out of 33 diseases/traits at 5% alpha compared to sex-homogeneous matched SNPs (*empP* < 0.001), and were significantly overrepresented in muscle, skeletal and stem cell development processes, and in calcium channel and microtubule complexes (*FDR* < 0.05, *empP* < 0.05). Interestingly, we found that *sex-het SNPs* significantly map to predicted expression quantitative trait loci (*Pr-eQTLs*) across brain and other tissues, methylation quantitative trait loci (*meQTLs*) during development, and transcription start sites, compared to *sex-homogeneous SNPs*. Finally, we verified that the sex-het disease/trait enrichment was not explained by *Pr-eQTL* enrichment alone, as sex-het *Pr-eQTLs* were more enriched than matched sex-homogeneous *Pr-eQTLs*. We conclude that genetic polymorphisms with sexually dimorphic effects on biometric traits not only contribute to fundamental embryogenic processes, but later in life play an outsized role in disease risk. These sex-het SNPs disproportionately influence gene expression and have a greater influence on disorders of body and brain than other expression-regulatory variation. Together, our data emphasize the genetic underpinnings of sexual dimorphism and its role in human health.

## Author summary

Risk for many diseases and related manifestations differs by sex. Here, we build on prior work to study a large set of anthropometric and biometric traits that could inform health differences by sex. We define a well-powered list of 2,320 sex-het SNPs showing sex-heterogeneity across multiple traits. We find that sex-het SNPs influence a large set of diseases and health-related traits. The sex-het SNPs are in/near genes with roles in skeletal and muscle development and are involved in regulating gene expression and DNA methylation, two important functions of the genome. We conclude that genetic variation with sexually dimorphic effects on biometric traits not only contributes to fundamental embryogenic processes but plays a role in disease risk later in life and is involved in the regulation of gene expression and epigenetic processes. Our results suggest that sex-heterogeneous SNPs link human physiology and pathology.

## Introduction

Most complex diseases show some degree of sex difference, in prevalence, manifestations, symptoms, comorbidities, and/or treatments and their side effects, requiring sex-personalized healthcare [1]. Biological differences between women and men are evident in physiology, yet genetic loci on the sex chromosomes have not yet explained sex differences in many heritable metabolic, autoimmune, and neuropsychiatric conditions and their underlying quantitative risk traits [2]. Potential autosomal origins of sex bias and the mechanisms by which the biology of sex may shape disease risk and outcomes are still not fully explored [3–5].

We previously hypothesized and tested several potential contributors to the genetics of sex bias in autism spectrum disorder (ASD) [6] and nine other complex diseases [7]. Using a novel approach to understanding sex differences in health via sexually-dimorphic physical traits, we identified autosomal SNPs showing sex-heterogeneity in their association with secondary sex characteristics (eight anthropometric measures) and tested the role of these SNPs in disease. We hypothesized that sex-heterogeneous (sex-het) SNPs, enriched for the biology of sexual dimorphism by definition, may contribute to disease biology, even if the anthropometric traits used to identify them appear unrelated to a disease of interest. Strikingly, we found that anthropometric sex-het SNPs (AH-SNPs) were enriched in association with all eight anthropometric measures, ASD, and 5 of 9 common, complex diseases, including some without sex differences in prevalence [6,7]. Our interpretation of this result was that the same mechanisms acting on secondary sex characteristic differences may influence disease risk through fundamental early developmental processes. However, the mechanisms by which sex-het SNPs act, their functions and roles in the genome, cells, and pathophysiology have not yet been clarified.

Here, we follow up our previous observation with improved detection power for sex-het SNPs within and across 20 biometric traits, thanks to publicly available summary statistics from UK Biobank and GIANT consortium. To characterize the role of sex-het SNPs, we investigate 1) enrichment in disease/trait association signal to confirm the importance to human health, 2) overrepresented biological processes to identify pleiotropic mechanisms, and 3) regulatory element overlap to identify specific genomic functions. We ultimately generate hypotheses about the role of biometric sex-het SNPs in physiology and disease. Our work contributes to clarifying autosomal mechanisms involved in sex differences across complex phenotypes, in order to advance our understanding of sex differences in health.

## Materials and methods

### Datasets

We downloaded sex-specific genome-wide summary statistics (Table 1) from Genetic Investigation of ANthropometric Traits (GIANT) consortium (S1 File) for 12 quantitative anthropometric traits: height and weight (2013), body mass index (BMI), hip circumference (HIP), BMI-adjusted HIP (HIPadjBMI), waist circumference (WC), BMI-adjusted WC (WCadjBMI), waist-hip ratio (WHR), BMI-adjusted WHR (WHRadjBMI) (2015), BMI adjusted for physical activity (BMIadjPA), BMI-adjusted WHR adjusted for physical activity (WHRadjBMIadjPA), BMI-adjusted WC adjusted for physical activity (WAISTadjBMIadjPA) (2017). We downloaded UK Biobank sex-specific genome-wide summary statistics from Neale lab (S1 File) for eight additional anthropometric and biometric traits: basal metabolic rate, body fat percentage, forced expiratory volume (FEV1), forced vital capacity (FVC), peak expiratory flow (PEF), systolic blood pressure (SBP), diastolic blood pressure (DBP), weight (Table 1). As reported in a meta-analysis of BMI and height traits between UK Biobank and GIANT studies [8], the two studies might partially overlap. The authors concluded that the overlap is small and likely has minimal effect. However, we have performed multivariate analysis across UK Biobank and GIANT with METAL that implemented–*overlap ON* option to ensure the meta-analysis was robust to overlapping samples (see below). The datasets from GIANT consortium included about 2.7M SNPs and up to 171,977 females and 152,893 males. The datasets from UK Biobank included ~13.8M SNPs and up to 193,627 females and 166,489 males.

**Table 1:** Sex-specific GWAS and genomic inflation factor (lambda) for Cochran’s Q.

**GIANT**	**genomic inflation factor**
Height	0.780
Weight	0.818
BMI	0.780
BMI adj PA	0.890
HIP	0.861
HIPadjBMI	0.849
WC	0.842
WCadjBMI	0.898
WHR	0.912
WHRadjBMI	0.939
WHRadjBMI adj PA	0.968
WAISTadjBMIadj PA	0.939
**UK Biobank**	**genomic inflation factor**
Basal metabolic rate	1.039
Body fat percentage	1.048
FEV1	1.018
FVC	1.016
PEF	1.022
Systolic Blood Pressure	1.017
Diastolic Blood Pressure	1.029
Weight	1.034

Abbreviations: BMI: body mass index; adj = adjusted for; PA: physical activity; WC: waist circumference; WHR: waist hip ratio; FEV1:forced expiratory volume; FVC: forced vital capacity; PEF: peak expiratory flow rate.

Genomic inflation factor for Cochran’s Q.

### Trait-specific sex-het SNPs

As a genome-wide implementation to assess differences in effect between males and females for each SNP within each biometric trait, we combined male-specific summary statistics with female-specific summary statistics with the fixed-effects meta-analysis commands (e.g., meta-analysis of Female BMI + Male BMI) in METASOFT (S1 File). However, instead of the meta-analysis trait association results, we extracted the marginal effects and the Cochran’s Q test of heterogeneity[9] to assess the sex differences in SNP effects on each trait. We observed little inflation for this statistic (lambda median = 0.94, Table 1). For each trait, we defined sex-het SNPs as those meeting *Cochran test P*_*Q*_ < 5x10^-8^ and MAF > = 0.1%. We compiled the sex-het SNPs from each trait, and we extracted a set of SNPs in low linkage disequilibrium (*r*^*2*^ < 0.2*)* with differential contribution to females and males for each trait.

### Multi-trait sex-het SNPs

To determine sex-heterogeneity estimates across 20 biometric traits and to increase the statistical power of the univariate analysis, we planned to use METAL software (below), which requires β_het_ and SE_het_ as input_._ Thus, we estimated a heterogeneity Z-score for each SNP and each trait based on the METASOFT output, where Z_het_ = (β Female– β Male) / sqrt(var(β Female) + var(β Male)). For each SNP, we converted Z_het_ to β_het_ and SE_het_: β_het_ = Z_het_ / sqrt(2p(1− p)(n + Z_het_^2)) and SE_het_ = 1 / sqrt(2p(1− p)(n + Z_het_^2)) where p is the allele frequency; N is the sample size (number of males + number of females). Note that *P*-values from Cochran’s Q and Z_het_ tests are highly correlated (median rho = 0.87).

We performed a meta-analysis across 20 traits using METAL (S1 File), as it is optimized to perform cross-trait analysis, including the option *overlap ON* for potential overlapping samples across GIANT and UK Biobank. Input included β_het_, SE_het_ along with the corresponding *P*_*Q*_ and the direction of the effect: positive if |β_female_| > |β_male_|, and negative if |β_female_| < |β_male_|. We applied the MAF > 0.001 cutoff and *P* < 5x10^-8^ significance threshold to the results of the multivariate meta-analysis, and we extracted a set of non-redundant SNPs. For each significant SNP from the multivariate analysis, we verified the results of the univariate analysis for each biometric trait, and we extracted the leading trait (minimum heterogeneity *P*-value) and the corresponding best *Cochran test P*_*Q*_. We combined the univariate and multivariate significant sex-het SNPs across biometric traits, and we performed a clumping LD analysis in PLINK (option–clump; S1 File) using the best *Cochran test P*_*Q*_ for each SNP. We extracted a final set of sex-het independent tagging SNPs within and across biometric traits in low linkage disequilibrium (*r*^*2*^ < 0.2*)* (S1 Table). Additionally, when the female absolute value marginal effect (beta estimate) of a given female SNP on our sex-het list was greater than the absolute value marginal effect of the same SNP in males, we tagged the given SNP as *female-driven sex-het SNPs* and *vice versa* when larger beta was observed in males (S1 Fig).

### Empirical P value

We assessed this set of biometric sex-het SNPs for enrichment of genetic signal compared to permuted lists of SNPs equally associated with biometric traits, but not sexually dimorphic, using similar methods for permuted sets as testing the sex-het sets of interest. We sampled 1,000 sets of random *sex-homogeneous SNPs*. We excluded the sex-het SNPs and all the SNPs in linkage disequilibrium (*r*^*2*^ *> 0*.*2*) with them, matching the allele frequency (+/- 0.001 for MAF<0.01, +/- 0.02 for MAF>0.01) and the combined-sex marginal effect (+/-75 positions in a ranked list) of the leading sex-het trait for validation with empirical *P*-values (*empP*). We used 100 (or 1,000 random sets to better refine the p-values) based on the complexity of the analyses described below, setting the significance thresholds at *empP = 0*.*05*. We compared the minor allele frequency distribution of the sex-het SNPs to the median MAF of UKBB traits and to the median of random *sex-homogeneous SNP* sets (S2 Fig).

### Enrichment of sex-het SNPs in disease and trait association signals

To identify pleiotropic effects between sex-het SNPs and disease, we assessed the enrichment of the sex-het SNPs in a large set of disease and trait association results. We downloaded publicly available summary statistics (S1 File and Table 2) for N = 16 diseases regardless of sex bias in prevalence: Alzheimer’s diseases (late onset), adult-onset asthma, anorexia nervosa (AN), chronic kidney disease (CKD), lacunar stroke, heart failure, post-traumatic stress disorder (PTSD), attention deficit hyperactivity disorder (ADHD), autism spectrum disorders (ASD), bipolar disorder (BIP), major depressive disorder (MDD), schizophrenia (SCZ), and cross-disorder association with 5 neuropsychiatric disorders, Tourette syndrome (TS), type 2 diabetes (T2D), insomnia. We did the same for N = 17 quantitative traits regardless of mean sex differences: alcohol use disorders identification test (AUDIT), age at completed education, age at first birth, automobile speed propensity, dietary fat intake, educational attainment, intelligence quotient (IQ), number of sexual partners, neuroticism, overall health rating, risk-taking behavior, total cholesterol, triglycerides, HDL cholesterol, LDL cholesterol, fetal own birthweight, maternal fetal birthweight. We extracted the overlapping SNPs between the set of sex-het SNPs and summary statistics for each disease/trait. Then, we calculated the percentage of overlapping SNPs at *P*<0.05 and tested whether it was significantly different from the null expectation of 5%. We validated the results by applying the same methods to 1,000 random sets of sex-homogeneous SNPs and estimating an empirical p-value based on the sets for which a greater proportion of overlapping SNPs show *P*<0.05 association compared with the observed sex-het SNPs (*empP*) (Table 2).

**Table 2:** Enrichment of biometric trait sex-heterogeneous SNPs in summary statistics for N = 16 diseases and N = 17 human phenotypes.

Diseases	study	N overlapping sex-het SNPs	% enrichment sex het SNPs	E/O	EmpP^
ADHD	Demontis 2019[34]	1124	7.93	56.2/89	0.11
Adult onset asthma	Ferreira 2019[35]	1405	7.62	70.3/107	0.027
Anorexia nervosa	Watson 2019[36]	1169	8.30	58.5/97	0.027
ASD	Grove 2017[37]	1323	7.18	66.2/95	0.044
BIP	Mullins 2021[38]	1307	9.042M*	65.4/118	0.096
CKD	Wuttke 2019[39]	1625	7.20	81.3/117	NS
Cross psychiatric disorders	Lee 2019[40]	1083	12.74	54.2/138	0.001
Heart failure	Shah 2020[41]	1323	7.25F*	66.2/96	0.014
Insomnia	Jansen 2019[42]	1533	7.50	76.7/115	0.079
Lacunar stroke	Traylor 2020[43]	1182	6.52	59.1/77	0.15
Late-onset Alzheimer’s	Kunkle 2019[44]	1523	5.32	76.2/81	NS
MDD	Wray 2018[45]	1786	5.48	89.3/98	NS
PTSD	Nievergelt 2018[46]	1400	6.43	70.0/90	NS
PGC-SCZ	See S1 File	1291	13.78	64.6/178	<0.001
Tourette syndrome	Yu 2019[47]	1349	5.49	67.5/74	NS
Type 2 diabetes	Xue 2018[48]	732	13.27	36.6/97	0.008
UKBB-Age at completed education	See S1 File	2312	8.22	115.6/190	0.005
Age at first birth	Barban 2016[49]	673	8.62	33.7/58	0.011
AUDIT	Sanchez-Roige 2018[50]	2285	6.30	114.3/144	NS
Automobile speed propensity	Karlsson-Linner 2019[51]	2103	7.75F*	105.2/163	0.020
Dietary fat intake	Meddens 2021[52]	2105	5.84	105.3/123	NS
Educational attainment	Lee 2018[53]	1784	13.85	89.2/247	<0.001
Intelligence quotient (IQ)	Savage 2018[54]	1529	11.34	76.5/173	0.003
N sexual partners	Karlsson-Linner 2019[51]	2103	8.99	105.2/189	<0.001
Neuroticism	Turley 2018[55]	1595	7.46M*	79.8/119	0.029
UKBB-Overall health rating	See S1 File	2312	9.86	115.6/228	<0.001
Risk behavior	Karlsson-Linner 2019[51]	2103	7.23	105.2/152	0.075
Total cholesterol	Willer 2013[56]	670	7.61	33.5/51	0.001
Triglycerides	Willer 2013[56]	669	7.47	33.5/50	0.005
HDL cholesterol	Willer 2013[56]	670	7.61	33.5/51	0.005
LDL cholesterol	Willer 2013[56]	669	6.87	33.5/46	0.019
Fetal own birthweight	Warrington 2019[57]	2172	7.92	108.6/172	0.066
Maternal fetal birthweight	Warrington 2019[57]	2126	7.99	106.3/170	0.009

* Chi square test P< = 0.05

^empirical p-value estimated on 1000 random set

Abbreviations: E/O expected and observed based on 5%

### Overrepresentation of sex-het SNPs in biological processes

We annotated genes to each sex-het SNP using ANNOVAR (S1 File). When the SNP was in UTR regions, splicing sites, exonic regions, or intronic regions, we assigned the corresponding gene. For intergenic SNPs, ANNOVAR assigned the two closest genes in both directions. To help determine a reasonable distance cutoff, we used our observation that the number of SNPs that are within 25kb from the nearest gene_A_ is 2.4x-11x greater than the number of SNPs with the further gene_B_ within 25kb. For greater distance (25-50kb, 50-100kb, 100-200kb, 200-500kb, and 500-2000kb), the enrichment in gene_A_ vs. gene_B_ distance rapidly decreased. We thus selected 25kb as a cut-off and we assigned the closest gene_A_ to each intergenic sex-het SNP when the SNP and gene_A_ were within 25kb, and we did not assign any gene to intergenic SNPs when the distance from the nearest gene and the SNP was > 25kb to reduce noise in our downstream gene-based analyses. We combined the list of corresponding genes (S2 Table) and the nearest genes assigned to proximal intergenic sex-het SNPs into our final sex-het gene list. We compared the resulting gene list to the products of published methods MAGMA [10] (35kb upstream, 10kb downstream) and FUMA GENE2FUNC [11] and provide Table 3A and 3B in S3 Table of alternative gene lists.

We used our sex-het gene list to perform overrepresentation analysis (ORA) in Gene Ontology (GO) pathways, biological processes, and cellular components using PANTHER (S1 File). We extracted the nominally significant results (FDR< 0.05). We performed the same analysis for 100 sets of permuted sex-homogeneous SNPs and sex-homogeneous gene lists derived with the same parameters. We used sex-homogeneous gene lists to calculate empirical p-values (*empP*) (Table 3 and S4 and S5 Tables).

**Table 3:** Enriched GO biological processes and cellular components in biometric trait sex-heterogeneous mapping genes (in/within 25kb distance) using ORA.

GO Biological process	N genes in human genome reference	N genes assigned to sex-het SNPs	expected number of genes	dir	FE	FDR	EmpP^#^
protein-DNA complex assembly (GO:0065004)	254	1	14	-	0.07	9.1x10^-3^	0.01
muscle structure development (GO:0061061)	460	51	25.35	+	2.01	4.6x10^-3^	0.01
muscle cell differentiation (GO:0042692)	235	29	12.95	+	2.24	4.3x10^-2^	0.01
exocytic process (GO:0140029)	67	13	3.69	+	3.52	4.7x10^-2^	0.01
protein-DNA complex subunit organization (GO:0071824)	294	3	16.2	-	0.19	3.9x10^-2^	0.02
stem cell differentiation (GO:0048863)	154	22	8.49	+	2.59	4.3x10^-2^	0.02
skeletal system development (GO:0001501)	471	50	25.96	+	1.93	1.1x10^-2^	0.03
*adaptive immune response (GO*:*0002250)*	*649*	*14*	*35*.*77*	*-*	*0*.*39*	2.1x10^-2^	*0*.*07*
*negative regulation of cell migration (GO*:*0030336)*	267	34	14.71	+	2.31	1.1x10^-2^	*0*.*08*
**GO Cellular component**							
voltage-gated calcium channel complex (GO:0005891)	17	7	0.94	+	7.47	5.6x10^-3^	0.01
calcium channel complex (GO:0034704)	26	8	1.43	+	5.58	8.7x10^-3^	0.01
dynein complex (GO:0030286)	62	11	3.42	+	3.22	3.0x10^-2^	0.01
microtubule associated complex (GO:0005875)	120	16	6.61	+	2.42	4.8x10^-2^	0.01
cellular_component (GO:0005575)	11293	689	622.36	+	1.11	6.3x10^-3^	0.01
cellular anatomical entity (GO:0110165)	11122	680	612.94	+	1.11	6.4x10^-3^	0.01
*glutamatergic synapse (GO*:*0098978)*	17	7	0.94	+	7.47	6.0x10^-3^	*0*.*06*

#Empirical p-value estimated on 100 random sets

### Enrichment of sex-het SNPs in gene sets

We performed enrichment analysis in estrogen and androgen responsive gene sets, previously analyzed [6,7]. Briefly, the androgen-responsive (AR) gene list was selected from Androgen Responsive Gene Database (ARGDB) for a total of 2,613 genes of which 2,500 matched the inclusion criteria. An estrogen-responsive (ER) gene list was selected from Estrogen Responsive Genes Database (ERGDB), with a total of 1,384 genes of which 1,148 matched the inclusion criteria [12,13]. We previously found enrichment in ER and AR gene sets only in four diseases by sex [7]. These two databases have not been updated from our previous publication. We calculated the proportion of overlap within the gene sets, and we estimated the empirical p-value using 100 permuted SNP sets as described above (results not shown).

### Enrichment of sex-het SNPs in regulatory regions

To assess whether the sex-het SNPs show regulatory function, we assessed the overlap with a set of 50 baseline annotations of regulatory elements publicly available (S1 File). We calculated the proportion of overlap within the regulatory elements for each category and we estimated the empirical p-value using 100 permuted SNP sets, as described above. The results for baseline annotations reported in Finucane et al. [14] are shown in S6 Table.

### Enrichment of sex-het SNPs in meQTLs

To assess the overlap of biometric trait sex-het SNPs with genetic variants affecting methylation (*meQTLs*) at fundamental timepoints for development, we downloaded a comprehensive genome-wide *cis* and *trans meQTL* longitudinal analysis in cord blood DNA and maternal blood during pregnancy of participants in the Avon Longitudinal Study of Parents and Children (ALSPAC) [15]. We performed enrichment analysis to assess the overlap between sex-het SNPs and unique *meQTLs* (P<1x10^-14)^ as described in Gaunt *et al*. [16]. We assessed the number of unique CpG probes for each sex-het *meQTL* SNP. We estimated an empirical p-value (*empP*) using 1,000 random matched SNP sets as described above (Table 4).

**Table 4:** Overlap between sex-heterogenous SNPs and (A) SNPs influencing DNA methylation (meQTLs), and (B) elastic-net predicted SNPs influencing gene expression across 49 tissues, 13 brain tissues (Pr-eQTLs).

**A. meQTLs**	**sex-heterogenous SNPs**	**% Overlap**	**EmpP#**	**N probes**	**EmpP#**	**range probes per meQTL SNP**
Cord blood	134	5.8	0.001	275	<0.001	1–21
Maternal blood	156	6.7	0.001	358	<0.001	1–24
**B. Pr-eQTLs**	**sex-heterogenous SNPs**	**% Overlap**	**EmpP** **#**	**N eGenes**	**EmpP** **#**	**range eGenes per Pr-eQTL SNP**
Across tissues	505	21.76	<0.001	1706	0.106	1–29
Brain tissues	264	11.37	0.001	598	0.017	1–6

#Empirical p-value estimated on 1000 random set

### Enrichment of sex-het SNPs in SNPs predicting gene expression under elastic-net model

We downloaded SNPs predicting gene expression in 49 tissues in GTEx (version 8) under an elastic-net variable selection model using PrediXcan (S1 File). Then, we extracted the proportion of overlap between the sex-het SNPs and the SNPs predicting genetically-regulated expression of genes (eGenes) across tissues and across brain tissues under an elastic-net model (from here *Pr-eQTLs*) and we compared the proportions derived by the same analysis of 1,000 sets of sex-homogeneous SNPs to estimate the empirical p-value (*empP*) (Table 4). We performed ORA on the set of eGenes regulated by sex-het *Pr-eQTL* SNPs across brain tissues (S7 Table). To assess whether the significant and suggestive enrichment in association signals for diseases/traits is driven by gene expression, we re-calculated the sex-het enrichment among the subset of association signals for SNPs predicting gene expression. We validated the analysis assessing the empirical p-value after creating 1,000 sets of permuted sex-homogenous SNPs that are also *Pr-eQTLs* matching the frequency of the sex-het SNPs (S8 Table).

## Results

**Fig 1:**
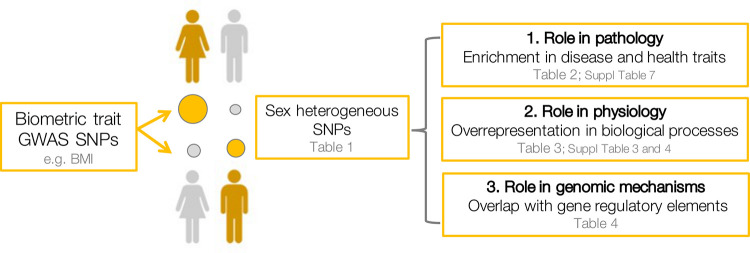
Outline of the presented analyses. We selected a set of sex-heterogeneous SNPs differentially influencing biometric traits. 1) Enrichment of sex-het SNPs in quantitative risk factors and diseases to define a role of sex-heterogeneity in physiology and pathology. 2) Overrepresentation analysis (ORA) of proximal genes assigned to sex-het SNPs in pathways, biological processes and cellular components. 3) Overlap of sex-het SNPs with regulatory elements, predicted eQTLs, and meQTLs.

Our overall study design was based on definition of sex-heterogeneous SNPs that act differently on males and females across biometric traits. First, we assessed whether these SNPs have an outsized role on pathology with a survey of common disease and health-relevant traits. Next, we assessed overrepresented functional properties of the genes associated with these SNPs to determine common physiology. Finally, we assessed genomic roles of the defined SNPs to identify mechanisms of action. Our study design is summarized in Fig 1.

### Biometric trait sex-heterogeneous SNPs

We previously found that SNPs having suggestively sexually-dimorphic association with anthropometric traits (AH-SNPs, *P*_*Q*_ < 0.0001) were relevant for ASD as well as other complex diseases and clinically-relevant quantitative traits [6,7]. To follow up this observation, we first wanted to identify an updated and more powerful set of sex-het SNPs. We expanded our approach to consider all measurable (biometric) traits, and we leveraged 12 sets of recent GIANT consortium sex-specific genome-wide summary statistics (Table 1). The female sample size is up to 171,977 and the male sample size is up to 152,893 individuals. Then, we took advantage of well-powered UK Biobank sex-specific genome-wide summary statistics from Neale lab (S1 File) to include 8 additional biometric traits analyzed on up to 193,627 females and 166,489 males, such as basal metabolic rate, body fat percentage, forced expiratory volume (FEV1), forced vital capacity (FVC), peak expiratory flow (PEF), systolic blood pressure (SBP), diastolic blood pressure (DBP), weight (Table 1). We applied two complementary analysis approaches: 1) within trait (univariate analysis) sex-heterogeneity and 2) multiple trait meta-analysis of sex-heterogeneity (multivariate analysis) to extract the set of sex-het SNPs (see Materials and Methods).

First, for each of the 20 traits, we applied a heterogeneity test between female and male summary statistics and we identified 180 independent SNPs that showed heterogeneity of effects across sexes (*Cochran test P*_*Q*_ [4x10^-17^ - 5x10^-8^]), mostly in GIANT traits. Second, we applied a multivariate approach with the aim to power our discovery analysis to identify SNPs that did not pass the heterogeneity significance threshold set for the univariate analysis but show modest sex-heterogeneity across multiple traits. For each SNP, we estimated the extent to which female effects outsized male effects using *a heterogeneity Z score*. Then, we meta-analyzed the female-male heterogeneity scores and *P*_*Q*_ across 20 traits to extract sex-het SNPs (N = 9,680; *P*_*meta*_ < 5x10^-8^) across 20 biometric traits. Finally, we combined univariate and multivariate results and we compiled 2,320 independent (*LD r*^*2*^ *< 0*.*2*) *sex-het SNPs* within and across biometric traits (S1 Table).

Almost all the ultimate sex-het SNPs (2,314/2,320) met the significance threshold in the multivariate analysis (vs. 6/2,320 appearing trait-specific), primarily from UK Biobank (2,028/2,320). The leading traits with the maximum sex-heterogeneity for each SNP are reported in S1 Table. Sex-het SNPs mapped across the entire autosomal genome and were nearly equally distributed between SNPs with greater (absolute value) effects in females (49.2% female-driven sex-het SNPs) and males (50.8% male-driven sex-het SNPs) (S1 Fig). The majority were driven by an effect in only one sex (N = 1,550), with nearly all the remaining SNPs showing nominal effects in opposite directions (N = 757), rather than differences in magnitude of effect (N = 13).

### Sex-het SNPs are enriched in disease and trait association signals

To assess whether SNPs showing sex-het effects in biometric traits are relevant for disease and quantitative health-related traits, we surveyed 16 diseases and 17 traits and we calculated sex-het SNP enrichment (percent *P*<0.05) in association signals at alpha 5%. We found significant enrichment in 6 out of 16 diseases and 13 out of 17 traits ranging between 7.2% in ASD and late-onset asthma (*empP* < *0*.*04)* and 13.8% in both educational attainment and schizophrenia (*empP* < 0.001, Table 2) compared with the null expectation of 5%. When we performed the same analysis with the subset of sex-het SNPs showing greater effects in males or females, we found most showed no male-driven or female-driven sex het SNP enrichment. Only 4 phenotypes appeared to show sex-specific enrichment out of 66 sex-specific analyses: nominally greater male-driven sex-het SNP enrichment in neuroticism and bipolar disorder (*FE* = 1.5x, *Chi square test P* = 0.029), and greater female-driven SNP enrichment in automobile speed propensity and heart failure (*FE* = 1.4–1.6x, *Chi square test P* = 0.04; Table 2).

### Tolerance of haploinsufficiency

Sex-het SNPs map in/near 1,325 genes (at distance between 0 kb and 25kb; see Materials and Methods and S2 Table). Because most GWAS signal is regulatory, we assessed the genes annotated to sex-het SNPs for tolerance of haploinsufficiency (pLI) as a metric of their sensitivity to expression changes [17]. Compared to reported distributions in the ExAC database [17], the genes mapped to our sex-het SNPs show substantial enrichment in highly-constrained genes pLI>0.9 (22.5%, *P*<0.000013) and depletion in non-constrained genes pLI<0.1 (38.1%, *P*<0.00001), demonstrating the utility of our nearest-gene annotation (S2 Table).

### Sex-het SNPs are in/near genes overrepresented in musculoskeletal development, calcium signaling, and cell anatomy

Since biometric trait sex-het SNPs play a role in disease and risk traits, we aimed to assess whether sex-het SNPs are in/near genes clustering in specific pathways or physiological functions that might lead to insight about their properties. We performed GO pathway overrepresentation analysis (*ORA*) on 1,107 mapped genes out of 1,325 genes (excluding unmapped genes, such as RNA genes), and we compared the enrichment in pathways to the entire set of 20,595 human genes. We did not find significant enrichment in any of 167 tested pathways at *FDR < 0*.*05*.

Then, we analyzed *ORA* of 15,807 GO biological processes and we found 83 significant processes at *FDR < 0*.*05*. Out of these processes, seven were significantly underrepresented in genes assigned to sex-het SNPs and 76 were overrepresented. We performed the same analysis using random sets of MAF- and association-matched SNPs in order to account for expected enrichment in trait-associated SNPs (see Materials and Methods). We confirmed that sex heterogeneity is driving the underrepresentation for *protein-DNA complex subunit organization* (GO:0071824) and *assembly* (GO:0065004) (*FE = 0*.*07–0*.*2X*; *empP < = 0*.*02*), and the borderline significant *adaptive immune response* (*FE = 0*.*4X*; *empP = 0*.*07*), and the overrepresentation for 5 out of 76 enriched biological processes (*FE = 1*.*9–3*.*5X*): *exocytic process* (GO:0140029), *muscle cell differentiation* (GO:0042692), and *muscle structure development* (GO:0061061) at *empP < = 0*.*01*, *stem cell differentiation* (GO:0048863) at *empP < = 0*.*02*, and *skeletal system development* (GO:0001501) at *empP < = 0*.*03 (*Tables 3, S4, and S5*)*. The set of genes assigned to the 100 random SNP sets (13,788 genes), were slightly and significantly enriched (*FE = 1*.*04–1*.*23X*) in 42 out of 76 biological processes (*FDR < 0*.*05*), indicating that trait association signal sufficient to show a sex difference may be driving much of the enrichment compared to all human genes. We did not find enrichment of sex-het SNPs in GO hormone-related pathways and biological functions. We separately investigated the proportion of sex-het SNPs overlapping androgen and estrogen responsive genes (5.2% and 2.4% respectively) from experimental datasets that we previously analyzed [6,7], but we did not find significant enrichment compared to the sex-homogeneous SNPs (*empP* > 0.05).

Finally, we tested the overrepresentation of sex-het SNPs in 508 GO cellular components. We identified 27 cellular components showing overrepresentation of genes assigned to sex-het SNPs (*FDR < 0*.*05*). The set of genes assigned to the 100 random SNP sets (13,788 genes) were slightly but significantly enriched (*FE = 1*.*04–1*.*25X*) in 8 out of the 27 cellular components with FDR *< 0*.*05*. Six out of the 27 showed significant empirical p-values: *voltage-gated calcium channel complex* (GO:0005891; *FE = 7*.*5X*), *calcium channel complex* (GO:0034704; *FE = 5*.*6X*), *dynein complex* (GO:0030286; *FE = 3*.*2X*), *microtubule associated complex* (GO:0005875; *FE = 2*.*4X*), *cellular component* (GO:0005575; *FE = 1*.*1X*), *cellular anatomical entity* (GO:0110165; *FE = 1*.*1X*), (*empP < = 0*.*01*), and *glutamatergic synapse* (GO:0098978; *FE = 7*.*5X*, borderline significant *empP = 0*.*06)* (Table 3).

### Sex-het SNPs overlap regulatory elements, eQTLs and meQTLs

We next characterized the genomic functional roles of the sex-het SNPs. First, we analyzed the overlap between the set of sex-het SNPs and N = 50 categories of regulatory elements as previously described (S1 File and S6 Table). Compared to sex-homogeneous permuted SNPs, sex-het SNPs nominally significantly overlap *transcription starting site (TSS; overlap = 2*.*63%*, *empP* < 0.05) [18] but not similarly powered regions such as enhancers, chromatin peaks [19], transcription factor binding sites (*TFBS*) or *CTCF* regions [18] (*empP* > 0.5; S6 Table). Strikingly, twenty categories (40%) were significantly depleted in sex-het SNPs compared to sex-homogeneous SNPs (*empP*_*sex-hom*_ < 0.05; S6 Table).

Since trait-associated variants from genome-wide association studies tend to overlap with expression quantitative trait loci, are more likely to be associated with gene expression [20,21], and have been shown to regulate DNA methylation [22], we hypothesized that sex-het SNPs extracted from large studies may be involved in the regulation of gene expression and DNA methylation. Interestingly, we found a small but significant proportion of unique sex-het SNPs overlapping genetic variants highly associated (*P* < 1x10^-14^) with CpG sites influencing DNA methylation (*meQTLs*) [22] (5.8% and 6.7% sex-het SNPs intersecting *meQTLs* in cord blood and maternal gestational blood, respectively; *empP* = 0.001, Table 4), previously described in Gaunt et al, 2016. We also found that 505 (21.8%) sex-het SNPs overlap with SNPs predicted to regulate gene expression under an elastic net model (*Pr-eQTLs*) across 49 tissues. Surprisingly, 264 (11.4%) sex-het SNPs overlap with *Pr-eQTLs* across 13 brain tissues. The cross-tissue and cross-brain enrichment was significant compared with enrichment of matched sex-homogeneous SNPs with equivalent trait-association (median = 17% and 9.6% respectively; *empP* < 0.001; Table 4). Male-driven and female-driven sex-het SNPs were equally distributed in enriched *Pr-eQTLs*. When excluding brain tissues from the cross-tissue analysis we found 468 (20.2%) sex-het *Pr-eQTLs*, indicating that most sex-het *Pr-eQTLs* across brain tissues influence gene expression in at least one other tissue. The sex-het *Pr-eQTLs* predict the gene expression of 1,706 unique eGenes across 49 tissues (N_sex-hom_ = 1292–1906 eGenes, median_sex-hom_ = 1590 eGenes; *empP = 0*.*11*) and 598 unique eGenes across brain tissues (_Nsex-hom_ = 374–654 eGenes, median_sex-hom_ = 504 eGenes; *empP = 0*.*017*). Sex-het *Pr-eQTLs* regulate up to 6 unique eGenes across brain tissues.

Out of the 459 eGenes regulated by sex-het SNPs across brain tissues represented in GO, only 35 overlapped with the 1,325 genes near/in sex-het SNPs by proximity and thus provided a semi-independent gene list. We performed ORA on this functionally-defined gene list, and 47 eGenes (3 also included in 1,325 gene list) showed almost 2-fold enrichment in endomembrane components for exchange and communication between cells (*FDR < 0*.*05;* S7 Table).

Across the diseases and phenotypes significantly or borderline significantly enriched in sex-het SNPs (26 out of 33; Table 2), we tested the enrichment in association signals across the subset of *sex-het Pr-eQTL SNPs* compared to the subset of *sex-homogeneous Pr-eQTL* SNPs to determine whether the enrichment was driven by the genetic function of the SNPs or their sex-heterogeneous properties. We found that 13 out of 26 disease/trait-association signals are enriched in cross-tissue sex het *Pr-eQTL* SNPs and 5 out of 13 also in cross-brain sex-het *Pr-eQTL* SNPs, compared to matching permuted sex-homogenous tissues/brain *Pr-eQTL* SNPs, mostly for neuropsychiatric traits and interestingly for total cholesterol, with ASD and AN showing borderline association (S8 Table). Given the small overlap, we did not test the subset of *meQTL* sex-het SNPs for disease/trait enrichment.

## Discussion

In the present study, we expanded our previous findings that autosomal common genetic variants with sexually dimorphic effects on anthropometric traits (AH-SNPs) exceptionally contribute to common disease risk, including diseases without strongly sex-biased prevalence [6,7]. Starting from our previous observation, here we aimed to improve detection power by finding a reliable set of sex-heterogeneous SNPs across a large set of biometric traits and explore their functional roles.

First, we meta-analyzed a larger (and broader) set of sex-specific summary statistics than our previous study, 20 well-powered biometric traits from UK Biobank and GIANT cohorts. We obtained 2,320 independent sex-het SNPs, most of them showing significant heterogeneity across multiple traits from either GIANT and/or UK Biobank. Next, we characterized their role across three domains: 1) pleiotropy of mechanisms involving sex-het SNPs and influencing risk for health-related traits, 2) specific biological processes and cellular components showing sex-het SNP enrichment that may lead to clues about physiology of health dimorphisms, and 3) specific roles in the genome played by sex-het SNPs that may lead to insight about the intersection of genetic polymorphism with sex.

Our enrichment analysis confirmed pleiotropy of mechanisms in the roles of biometric sex heterogeneous SNPs influencing the biology of a large set of neuropsychiatric, cardiovascular and autoimmune diseases, self-reported characteristics, behavioral, and metabolic traits. We did not attempt to directly replicate our previous findings, rather we selected traits because well-powered summary statistics were available, and a variety of organs and systems were represented. We found enrichment of sex-het SNPs in association signals for diseases that show female bias (anorexia nervosa, asthma), male-bias (ASD, SCZ), and little bias (heart failure, type 2 diabetes) in prevalence. Diseases with no sex-het enrichment can show mild sex-bias in prevalence (e.g., lacunar stroke) or relatively strong sex-bias (e.g., ADHD and Tourette syndrome). We were not able to identify shared characteristics across the enriched diseases vs. non-enriched diseases, but the heterogeneity of genetic data and power across studies is a limitation for direct comparison. For example, the enrichment in cross-neuropsychiatric disorders [23] was driven by SCZ and ASD, however, previous analyses within the non-enriched BIP, MDD and ADHD showed more heterogeneity across cohorts than for SCZ and ASD [23]. More than 80% of the risk factor traits showed enrichment in sex-het SNPs, including both self-reported traits (e.g., overall health rate, educational attainment) and health-related traits (e.g., total cholesterol, IQ). We found nominal specificity of direction (male vs. female effects) in only 4 of 33 diseases and traits with no evident relationship between sex and trait, which could be consistent with our overall finding of the lack of relationship between sex-het enrichment and prevalence differences by sex, or could be a chance finding due to multiple testing, but in either case is difficult to interpret. Although quantitative traits show better statistical power than dichotomous diagnoses, these findings suggest a role for sex-het SNPs in physiology, likely acting during the human developmental stages.

Since sex-het SNPs showed an exceptional role across relevant human phenotypes, we next assessed the potential biological mechanisms involving biometric trait sex-het SNPs. In order to apply biological knowledge, we needed to map sex-het SNPs to genes. We annotated 57% sex-het SNPs with the corresponding mapping genes and/or the proximal genes (< 25kb distance). This annotation method was appealing due to its simplicity and prior evidence of the importance of proximal genes [24], however, incomplete knowledge of the relationship between associated SNPs and genes is a limitation of our study. Overall, we found strong overrepresentation of constrained genes (pLI>0.9), supporting the utility of our annotation (compared to other annotation approaches; Table 3A and 3B in S3 Table). Most of the gene ontology category overrepresentation we observed naively was also present in permuted sex-homogeneous SNPs, likely due to allele frequency biases and marginal effects enriched by our sex-het ascertainment, emphasizing the importance of our empirical assessment. We discuss only categories specific to sex-het SNPs below.

The gene sets with expression levels influenced by androgens and estrogens showed a small overlap, and they were not enriched in sex-het SNPs. Previously, we observed that AH-SNPs showed overlap with AR and ER datasets compared with permuted SNP lists (*P* < 0.01, each), although the amount of overlap was small [6]. Since we now include additional biometric traits rather than exclusively secondary-sex characteristics, the lack of hormone-driven enrichment could be due to broader trait ascertainment. Alternatively, we increased signal-to-noise by using more stringent sex-heterogeneity criteria and refined our matching of the permuted gene sets with the sex-het SNPs, so the technical changes may also have led to differing significance for enrichment and corrected a previous false positive result.

Sex-het SNPs were specifically enriched in important cellular components like calcium channels and cytoskeletal proteins, such as microtubule-dynein complexes. Interestingly, calcium channel related genes represented on our sex-het list include those important in skeletal muscle, cardiac, brain, and mitochondrial function. There are some known estrogen-responsive properties for cardiac and mitochondrial calcium channel activity [25]. Sex differences have also been observed in calcium channel blocker benefits [26]. But across the literature, evaluation of sex differences in calcium channel functions and health consequences is limited. Several of the calcium channel sex-het genes appear to be annotated with sperm motility (*CATSPER1*) [27] conditions or be involved in increase in neuronal firing in male central nervous system (*CACHD1*) [28]. Surprisingly, we found sex-het SNP enrichment in fundamental proteins like the cytoskeletal proteins that play a wide range of functional and structural roles in human cells, such as transport, hormone secretion and synaptic transmission [29]. In men, microtubules are vital for organelle transport and cellular divisions during spermatogenesis and sperm motility process [30]. In contrast, components of the assembly and organization of the protein-DNA complex were underrepresented, suggesting their importance in the body and conservation across sexes. Finally, genes assigned to sex-het SNPs were overrepresented in muscle, skeletal and stem cell development processes, suggesting that sex heterogeneity in response to genetic variation is influential from early stages of development, even if manifesting in health and biometric trait sex differences in adulthood.

Most of the regulatory regions tested using the baseline annotation [14] showed no enrichment in sex-het SNPs compared to permuted matched sex-homogeneous SNPs. In fact, a large proportion (40%) of tested categories were significantly underrepresented in sex-het SNPs, and further study might demonstrate the importance of cross-sex constraint in genome regulation. Only the overlap with transcription start sites (TSS) was nominally significant, suggesting that sex-het SNPs may affect gene expression, selection of transcriptional start sites, and transcript isoforms. TSS is the major contributor to tissue-specific regulation of gene expression and TSS choice may also vary across developmental stages or during cell differentiation [31]. However, the other regulatory annotations may have less accuracy and limit the power to detect enrichment, so it is difficult to interpret the specificity of this category to sex-het SNPs. Since the assessment of regulatory regions depends on accessibility, it is cell type- and condition-selective, with only a small fraction of all genome-encoded elements becoming actuated in a given cellular context [32]. Thus, further experimental investigations will be needed to refine the annotations and sex-het SNP enrichment.

We investigated in more depth whether sex-het SNPs may play a role in gene expression. Sex-het SNPs significantly overlapped genetic variants influencing DNA methylation variability in cord and maternal blood during pregnancy [16], two tissues that are fundamental for determining early fetal development. We did not assess other categories specific to methylation or epigenetic variability. Prior evidence showed that sparse polygenic models are a more effective approach than single-variant association analysis for prioritizing multiple causal *eQTL* variants at a single gene [33]. Thus, we tested SNPs predicting gene expression, and more than 20% of the sex-het SNPs (vs. 17% sex-homogeneous SNPs) significantly overlap with SNPs predicting gene expression across 49 tissues under an elastic-net prediction model and 11% (vs. 9.6% sex-homogeneous SNPs) also across brain tissues. The brain expression signal despite lack of brain biometric trait ascertainment, suggests that the enrichment we observe in psychiatric, behavioral and neurological traits (and potentially prominent sex differences in these traits) could result from pleiotropy in development across tissues. The sex-het *Pr-eQTLs* significantly regulate eGenes across brain tissues, of which a subset is overrepresented in endomembrane system components, particularly vesicular proteins involved in transport within the cell, early secretory pathway and in Golgi structure (S7 Table). Consistent with overrepresentation of the genes near sex-het SNPs, this observation suggests that fundamental processes for the anatomy and structure of cells are enriched in sex-heterogeneity. We found overlap between 30–50% sex-het *meQTLs* and sex-het *Pr-eQTLs* across tissues and across brain tissues, respectively, suggesting that these categories may capture the same biological signal. Finally, we re-assessed enrichment in human phenotypes for sex-het *Pr-eQTL* SNPs compared to the permuted matched sex-homogeneous *Pr-eQTL* SNPs and found that the enrichment in disease association is not explained by the genetic function of the SNPs but by the selection for sex-heterogeneity.

In conclusion, our results suggest that sex-heterogenous SNPs are involved not only in sexually dimorphic biometric traits but also contribute disproportionately to disease and health-related traits. Sex-het SNPs are near genes that during fundamental early stages of development will sex-differentially shape the structure of the body. Key cellular functions involved include calcium signaling and cell anatomical components. Sex-het SNPs map to regions critical for DNA methylation, transcription start sites and the regulation of expression of genes. Additional experimental investigations will allow a better understanding of the cell-dependent and state-dependent role of sex heterogeneous genetic variation in humans.

## Supporting information

S1 File List of URLs of tools and databases. (PDF)

S1 Fig Manhattan plots of female-driven and male-driven sex-heterogeneous SNPs. (TIFF)

S2 Fig Minor allele frequency distribution of the sex heterogeneous SNPs. (TIFF)

S1 Table Set of independent biometric trait sex-heterogenous SNPs from univariate and multivariate meta-analysis. (XLSX)

S2 Table N = 1,325 genes assigned to sex-heterogeneous SNPs within a distance of 25Kb. (XLSX)

S3 Table **S3A Table in S3 Table** Gene Prioritization by MAGMA—SNP2GENE **S3B Table in S3 Table**. Gene Prioritization by FUMA—SNP2GENE / GENE2FUNC.
(XLSX)

S4 Table Enrichment of sex-heterogenous SNPs mapping genes (in/within 25kb) in Gene Ontology Biological Processes. (XLSX)

S5 Table Enrichment of sex-heterogenous SNPs mapping genes (in/within 25kb) in Gene Ontology Cellular Components. (XLSX)

S6 Table Overlap between biometric trait sex-heterogeneous SNPs and baseline regulatory elements included in Finucane et al. 2013.
(XLSX)

S7 Table Cross-brain tissue eGenes regulated by sex-heterogeneous Pr-eQTL SNPs. (XLSX)

S8 Table Enrichment of biometric trait sex-heterogeneous SNPs in summary statistics for N = 16 diseases and N = 17 human phenotypes. (XLSX)
