# Supplementary material for: Sex-heterogeneous SNPs disproportionately influence gene expression and health
Source: PLoS Genet. 2022 May 5;18(5):e1010147. doi: 10.1371/journal.pgen.1010147 (PMC9070888; doi:10.1371/journal.pgen.1010147)
Supplement: S1 File — (PDF) [file pgen.1010147.s001.pdf]

URLs

METASOFT meta-analysis software - http://genetics.cs.ucla.edu/meta/

METAL meta-analysis software - https://genome.sph.umich.edu/wiki/METAL_Documentation

PLINK clumping software - https://www.cog-genomics.org/plink/

ANNOVAR annotation software - https://annovar.openbioinformatics.org/en/latest/

PANTHER - ORA software - http://www.pantherdb.org

Baseline annotations - https://storage.googleapis.com/broad-alkesgroup-public/LDSCORE/readme_baseline_versions

meQTLs - http://www.mqtldb.org

PrediXcan predicted gene expression software - https://github.com/hakyimlab/PrediXcan

Disease and health related trait GWAS for enrichment analysis:

Psychiatric Genomics Consortium (PGC) - https://www.med.unc.edu/pgc/download-results/

Adult-onset of asthma - https://genepi.qimr.edu.au/staff/manuelF/gwas_results/main.html

Chronic kidney disease (CKD) - http://ckdgen.imbi.uni-freiburg.de

Cardiovascular Disease Knowledge Portal - http://www.broadcvdi.org/

Insomnia - https://ctg.cncr.nl/software/summary_statistics

Late-onset Alzheimer disease - https://www.niagads.org/home

Type 2 diabetes - http://cnsgenomics.com/data.html

Age at completed education/ Overall health - http://www.nealelab.is/uk-biobank/ukbround2announcement

Sociodemographic traits - https://www.thessgac.org/data
Intelligence - https://ctg.cncr.nl

Global Lipids Genetics Consortium -
http://www.sph.umich.edu/csg/abecasis/public/lipids2013

Birthweight - https://egg-consortium.org/
