# Supplementary figures and images for: Sex-heterogeneous SNPs disproportionately influence gene expression and health
Source: PLoS Genet. 2022 May 5;18(5):e1010147. doi: 10.1371/journal.pgen.1010147 (PMC9070888; doi:10.1371/journal.pgen.1010147)

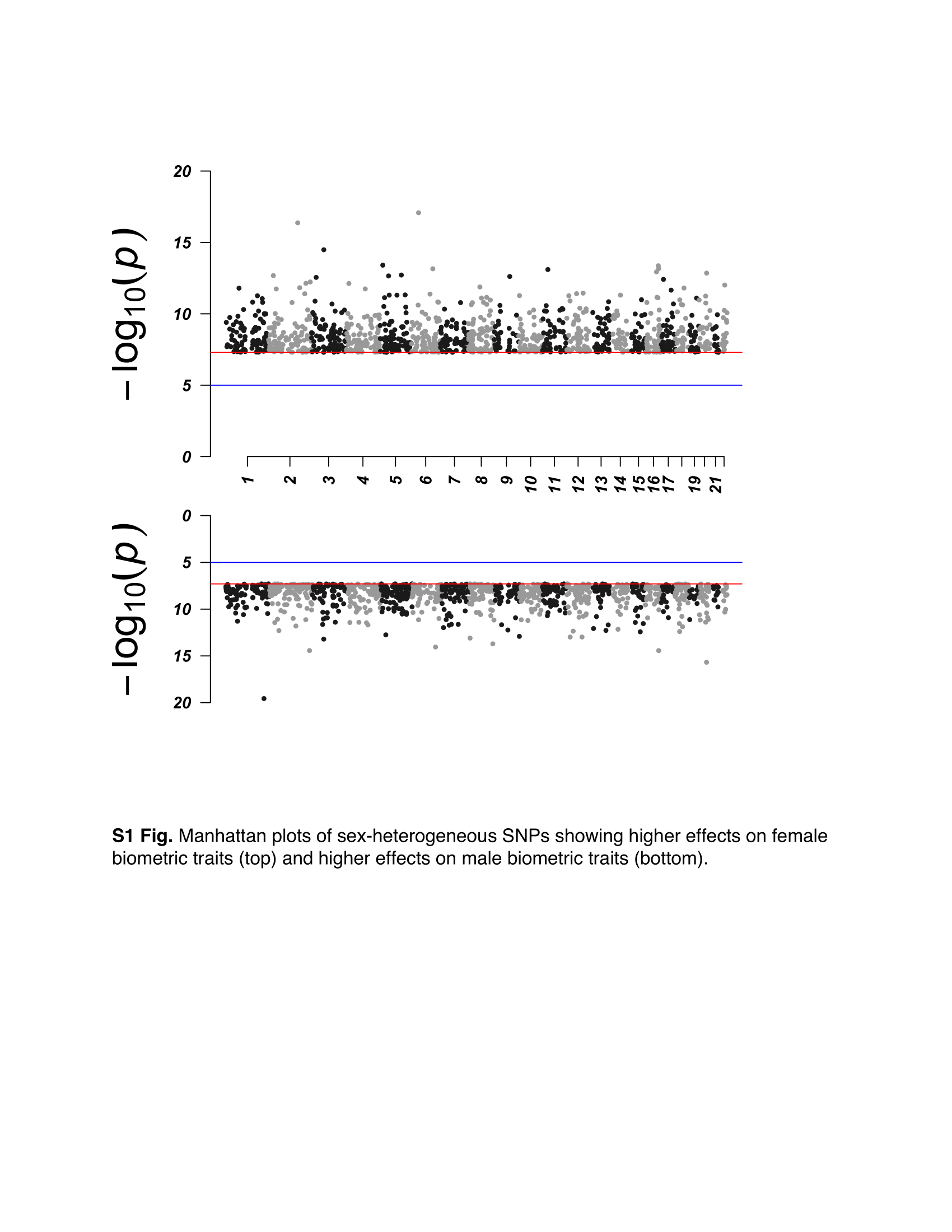

Supplement: S1 Fig — (TIFF) [file pgen.1010147.s002.tiff]

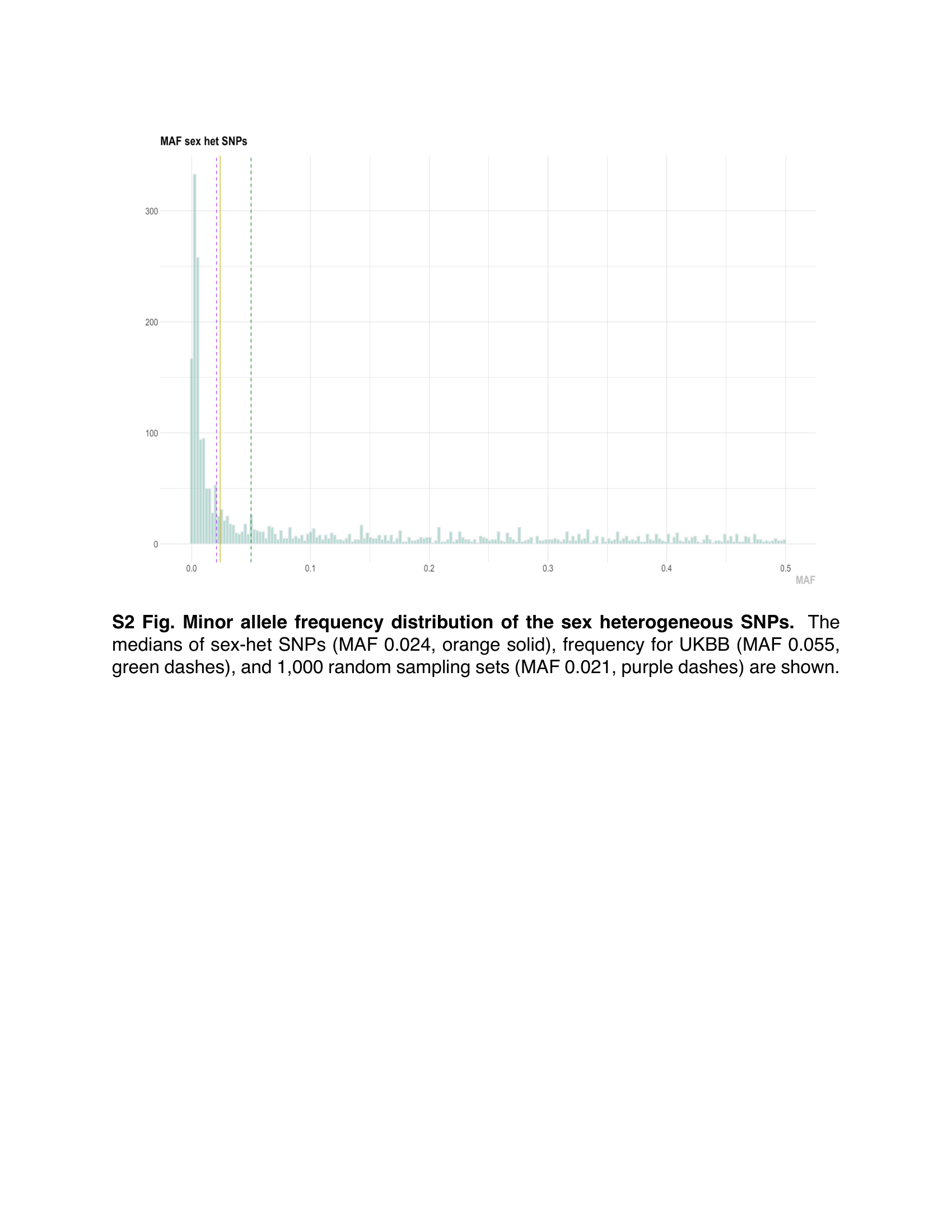

Supplement: S2 Fig — (TIFF) [file pgen.1010147.s003.tiff]
